# Processing mechanism of guanidinoacetate in choroid plexus epithelial cells: conversion of guanidinoacetate to creatine via guanidinoacetate N-methyltransferase and monocarboxylate transporter 12-mediated creatine release into the CSF

**DOI:** 10.1186/s12987-022-00328-w

**Published:** 2022-06-03

**Authors:** Ryuta Jomura, Shin-ichi Akanuma, Yoshiyuki Kubo, Masanori Tachikawa, Ken-ichi Hosoya

**Affiliations:** 1 Department of Pharmaceutics, Graduate School of Medicine and Pharmaceutical Sciences, University of Toyama, 2630 Sugitani, Toyama, 930-0194 Japan; 2 Graduate School of Biomedical Sciences, Tokushima University, 1–78–1 Shomachi, Tokushima, 770–8505 Japan

**Keywords:** Creatine, Guanidinoacetate (GAA), Monocarboxylate transporter (MCT/SLC16A), MCT12 (SLC16A12), Choroid plexus epithelial cells, Blood-cerebrospinal fluid barrier (BCSFB), Guanidinoacetate N-methyltransferase (GAMT)

## Abstract

**Background:**

Guanidinoacetate (GAA) induces epileptogenesis and neurotoxicity in the brain. As epileptic animal models have been reported to show elevated cerebral GAA levels, the processing mechanism of GAA in the brain is important for maintaining brain homeostasis. We have revealed that GAA in the cerebrospinal fluid (CSF) is removed by incorporation into the choroid plexus epithelial cells (CPxEpic), which form the blood-CSF barrier (BCSFB). However, the processing mechanism of GAA incorporated into CPxEpic remains unknown. We have reported that monocarboxylate transporter 12 (MCT12) functions as an efflux transporter of GAA and creatine, a metabolite of GAA, in the kidneys and liver. Therefore, we aimed to clarify the role of MCT12 in GAA dynamics in CPxEpic.

**Methods:**

Protein expression and localization in CPxEpic were evaluated using immunohistochemistry. Metabolic analysis was performed using high-performance liquid chromatography (HPLC) 24 h after the addition of [^14^C]GAA to TR-CSFB3 cells, which are conditionally immortalized rat CPxEpic. The efflux transport of [^14^C]creatine was evaluated in TR-CSFB3 cells after transfection with MCT12 small interfering RNA (siRNA). The CSF-to-brain parenchyma transfer of creatine was measured after intracerebroventricular injection in rats.

**Results:**

Immunohistochemical staining revealed that MCT12-derived signals merged with those of the marker protein at the apical membrane of CPxEpic, suggesting that MCT12 is localized on the apical membrane of CPxEpic. The expression levels of guanidinoacetate N-methyltransferase (GAMT), which catalyzes the conversion of GAA to creatine, in TR-CSFB3 cells was also indicated, and GAA was considered to be metabolized to creatine after influx transport into CPxEpic, after which creatine was released into the CSF. Creatine release from TR-CSFB3 cells decreased following MCT12 knockdown. The contribution ratio of MCT12 to the release of creatine was more than 50%. The clearance of CSF-to-brain parenchyma transfer of creatine was 4.65 µL/(min·g brain), suggesting that biosynthesized creatine in CPxEpic is released into the CSF and supplied to the brain parenchyma.

**Conclusions:**

In CPxEpic, GAA is metabolized to creatine via GAMT. Biosynthesized creatine is then released into the CSF via MCT12 and supplied to the brain parenchyma.

**Supplementary Information:**

The online version contains supplementary material available at 10.1186/s12987-022-00328-w.

## Background

Guanidino compounds, such as guanidinoacetate (GAA), γ-guanidinobutyrate (GBA), guanidinoethanesulfonate (GES), β-guanidinopropionate (GPA), guanidinosuccinate (GSA), α-guanidinoglutaric acid (GGA), N-acetylarginine, methylguanidine, homoarginine, and creatinine (CRN), have been reported to induce seizures [1, 2]. In addition, animal models of epilepsy have been reported to exhibit elevated GAA, CRN, and methylguanidine levels in the brain after convulsions [1]. In contrast, GAA is known to be a precursor of creatine, which is metabolized to CRN and methylguanidine [3]. Creatine is known to be essential for energy homeostasis in the brain as creatine deficiency induces cerebral creatine deficiency syndromes (CCDSs), which lead to mental retardation and language delay [4, 5]. As creatine biosynthesis and metabolism occur in the brain [3, 4], strict control of creatine dynamics is needed to maintain the balance between creatine and guanidino compounds that induce convulsions. Therefore, elucidation of the mechanisms maintaining the levels of guanidino compounds in the brain can lead to an understanding of cerebral energy homeostasis, in addition to the pathology of epilepsy and the regulation mechanism of seizures.

We have revealed that GAA and CRN were eliminated from the brain via the blood-cerebrospinal fluid (CSF) barrier (BCSFB), although these compounds are hardly eliminated through the blood–brain barrier (BBB) [6–8]. BCSFB restricts the non-selective intercellular permeation of compounds between the CSF and circulating blood via tight junctions formed by the choroid plexus epithelial cells (CPxEpic) [2]. Transcellular transport occurs via various transport systems for the permeation of compounds between the CSF and circulating blood across the BCSFB [3]. Elimination of CRN across the BCSFB is reportedly mediated by the organic cation transporter 3 [OCT3/solute carrier (SLC) 22A3] [7]. Since CRN is the end product of creatine metabolism [5], CRN is considered to be circulated through the blood flow and then get excreted into urine after elimination from the brain. In contrast, GAA is known not only as a convulsant, but also as a biosynthetic precursor of creatine. In addition, the mRNA expression of guanidinoacetate N-methyltransferase (GAMT), which catalyzes the metabolism of GAA to creatine [9], has been reported to be detected in the choroid plexus of the adult rats [10], although it is not detected in the fetal stage [11]. Furthermore, [^14^C]GAA was eliminated from the CSF after intracerebroventricular injection of [^14^C]GAA into the rats, and isolated choroid plexus incorporates extracellular [^14^C]GAA [6]. Thus, GAA in the CSF is considered to be partly incorporated into CpxEpic and could be used as a source of creatine, at least. In the transport processes, GAA is taken up by CpxEpic via the creatine transporter (CRT/SLC6A8) and taurine transporter (TauT/SLC6A6) [3, 6, 12]. However, the processing mechanism after the incorporation of GAA into CpxEpic remains unclear. As GAA is a biosynthetic precursor of creatine, it is possible that CpxEpic play a role in maintaining GAA and creatine levels in the brain by creatine provision.

Efflux transporters of GAA and creatine are considered to be important to understand the dynamics of GAA and creatine in CpxEpic. GAA and creatine are reported substrates for SLC6A and SLC16A family transporters [3, 13]. Creatine is a substrate for CRT, monocarboxylate transporter 9 (MCT9/SLC16A9), and MCT12 (SLC16A12) [3, 13, 14]. GAA is also recognized by CRT and MCT12 as a substrate [6, 13]. In addition, GAA is a substrate for TauT and γ-aminobutyric acid (GABA) transporters (GATs) [8]. SLC6A family transporters that recognize GAA and/or creatine as substrate(s) have been reported to be Na^+^- and Cl^–^-coupled symporters [3]. Since the concentrations of Na^+^ and Cl^−^ are much higher in the extracellular fluid than in the intracellular fluid, SLC6A transporters have been suggested to mediate the influx transport of substrates in non-excitable cells, such as CpxEpic. On the other hand, MCT9 and MCT12 have been reported to exhibit different transport mechanisms than SLC6A transporters. MCT9 has been suggested to be an H^+^/creatine exchanger [15]. As the extracellular pH is higher than the intracellular pH, MCT9 seems to function as an influx transporter [14]. In contrast, MCT12 acts as a facilitative transporter [3]. We have reported that MCT12 plays a role in the efflux transport of GAA and creatine from renal proximal tubular epithelial cells and hepatocytes, respectively, to the extracellular fluid of the blood side [13, 14]. Therefore, MCT12 may be a potential candidate for mediating the efflux transport of GAA or creatine from CpxEpic.

The purpose of this study was to elucidate the dynamics of GAA after incorporation into CpxEpic. To elucidate the possibility that MCT12 mediates efflux transport from CpxEpic, the localization of MCT12 protein in CpxEpic was determined by immunohistochemical analysis, which indicated that MCT12 was localized on the CSF side membrane of CpxEpic. The expression levels and activity of GAMT were observed in CpxEpic, and it was conceived that GAA is metabolized to creatine and then creatine is released into the CSF. Creatine transport was analyzed using TR-CSFB3 cells, which are conditionally immortalized rat CpxEpic [16]. This suggests that MCT12 contributes to the efflux of creatine from CpxEpic. After the in vivo injection of [^14^C]creatine into the rat lateral ventricle, transfer of the [^14^C]creatine to the brain parenchyma was observed. Consequently, it is suggested that GAA is converted to creatine in CpxEpic, and then biosynthesized creatine is released into the CSF and supplied to the brain parenchyma.

## Methods

### Animals

Male Wistar/ST rats (6 weeks old, 150–180 g) were purchased from Japan SLC (Hamamatsu, Japan). The animals were maintained in a controlled environment, and all experiments were approved by the Animal Care Committee of the University of Toyama.

### Reagents

Analytical-grade chemicals were used in this study. Creatine hydrate, [4-^14^C]-(2.11 GBq/mmol), and guanidinoacetic acid, [1-^14^C]-([^14^C]GAA, 2.04 GBq/mmol) were purchased from Moravek Biochemicals (Brea, CA, USA). Mannitol, D-[1-^3^H (N)]-(455 GBq/mmol) was purchased from PerkinElmer (Waltham, MA, USA).

### Immunohistochemical staining

Immunohistchemical staining was performed according to a previously reported protocol [14]. Frozen sections of the rat brain (20 µm thick) were prepared and immersed in 0.1% Triton-X100 dissolved in phosphate-buffered saline without Ca^2+^ and Mg^2+^ [PBS ( −); 137 mM NaCl, 8.1 mM Na_2_HPO_4_, 2.7 mM KCl, and 1.5 mM KH_2_PO_4_] for 1 h at room temperature. After the treatment with 10% goat serum for 1 h at room temperature, the sections were incubated overnight with guinea pig anti-MCT12 (3 µg/mL) [13], guinea pig anti-GAMT (3 µg/mL) [17], and/or mouse anti-Na^+^, K^+^-ATPase α1 (2 µg/mL; clone: C464.6; Merck, Darmstadt, Germany) antibodies. For antigen absorption, anti-MCT12 antibodies were incubated with PBS ( −) with or without the antigen peptide (4.36 µg/mL) for 6 h at 4 °C before the primary antibody reaction. The sequence of antigen peptides for anti-MCT12 antibodies was KEDPSGPEKSHDRDAQRED, which is a 200–218 amino acid sequence of rat MCT12 [National Center for Biotechnology Information (NCBI) reference sequence: NM_001191637.1]. The sections or cells were incubated with species-specific secondary antibodies labeled with Alexa Fluor 488 or Alexa Fluor 568 (Thermo Fisher Scientific, Waltham, MA, USA) for 2 h at room temperature. Confocal images were obtained using a confocal laser-scanning microscope (LSM780; Carl Zeiss, Oberkochen, Germany).

### Immunoblotting

Collection of the crude membrane fraction of TR-CSFB3 cells, sodium dodecyl sulfate–polyacrylamide gel electrophoresis (SDS-PAGE) and electroblotting were performed as previously described [13]. The protein samples separated by SDS-PAGE were electroblotted on polyvinylidene fluoride (PVDF) membrane (Amersham Hybond P PVDF 0.45; GE Healthcare, Chalfont St. Giles, UK). The membrane were incubated with a blocking solution (125 mM NaCl, 0.1% Tween-20, 1% non-fat dry milk, and 25 mM Tris–HCl, pH 7.4) for 1 h at room temperature and then treated with primary antibodies (1 µg/mL anti-MCT12 or 0.1 µg/mL anti-Na^+^, K^+^-ATPase α1 antibodies) for 2 h at room temperature. For antigen absorption, anti-MCT12 antibodies were incubated with blocking buffer with or without the antigen peptide (4.36 µg/mL) for 6 h at 4 °C before the primary antibody reaction. The PVDF membranes were then treated with horseradish peroxidase-conjugated anti-guinea pig or anti-mouse IgG antibodies for 2 h at room temperature. The signals were visualized and signal intensity was quantified using the ImageJ software (National Institutes of Health, Bethesda, MD, USA). Protein expression levels of MCT12 were normalized to those of Na^+^, K^+^-ATPase α1.

### Reverse transcription-polymerase chain reaction

RT-PCR analysis was conducted as described previously [13]. Total RNA was isolated from the liver and TR-CSFB3 cells, and cDNA was synthesized from 0.5 µg of total RNA as a template by reverse transcription. PCR was performed using specific primers for 35 cycles: 98 °C for 10 s, 60 °C for 30 s, and 72 °C for 1 min. The primer sequences used in this study are listed in Table 1.

**Table 1:** Primers used for gene expression analysis

Genes	Gene bank accession number	Orientation	Primer sequence (5′–3′)	Product size
*MCT12*	NM_001191637.1	Forward	AGCCTTCCTTCTTTGTGG	179 bp
		Reverse	TCTGATCTAACTCCTTCGC	
*GAMT*	NM_012793.2	Forward	TCTGACACGCACCTGCAGATCC	584 bp
		Reverse	GCATAGTAGCGGCAGTCGGCTG	
*β-Actin*	NM_031144.3	Forward	TCATGAAGTGTGACGTTGACATCCGT	285 bp
		Reverse	CCTAGAAGCATTTGCGGTGCACGATG	

### Metabolic analysis

Metabolic analysis was performed as described previously [14]. TR-CSFB3 cells were cultured in collagen type I-coated cell culture plates with high-glucose Dulbecco’s modified Eagle’s medium (DMEM) supplemented with 10% fetal bovine serum (v/v) at 33 °C. The cells were incubated with high-glucose DMEM containing 18.2 µM [^14^C]GAA for 24 h at 37 °C. Using the cell lysate of TR-CSFB3 cells, high-performance liquid chromatography (HPLC) was performed as described previously [14]. Typical chromatograms of [^14^C]creatine and [^14^C]GAA were obtained by HPLC analysis using intact [^14^C]creatine and [^14^C]GAA.

### Transport analyses

Transport analyses were conducted according to a previously reported protocol [14]. For [^14^C]creatine uptake, after washing TR-CSFB3 cells, the uptake reaction was initiated by replacing the medium with the transport solution (122 mM NaCl, 25 mM NaHCO_3_, 10 mM d-glucose, 3 mM KCl, 1.4 mM CaCl_2_, 1.2 mM MgSO_4_, 0.4 mM K_2_HPO_4_, and 10 mM HEPES–NaOH, pH 7.4) containing [^14^C]creatine (8.77 µM). After incubation at 37 °C for a designated period, the uptake reaction was terminated by removing the medium and washing the cells with ice-cold ECF buffer. The cell-to-medium ratio [Eq. (1)] was used to express the uptake of [^14^C]creatine.

1 $${\text{Cell/medium}}\,{\text{ratio}}\,\left( {\mu {\text{L/mg}}\,{\text{protien}}} \right) = \frac{{\left[ {{}^{14}{\text{C}}} \right]{\text{Creatine}}\,{\text{in}}\,{\text{the}}\,{\text{cells}}\,\left( {{\text{dpm/mg}}\,{\text{protien}}} \right)}}{{\left[ {{}^{14}{\text{C}}} \right]{\text{Creatine}}\,{\text{in}}\,{\text{the}}\,{\text{medium}}\,\left( {{\text{dpm/}}\mu {\text{L}}} \right)}}$$

For efflux transport, TR-CSFB3 cells were preincubated with high-glucose DMEM containing 8.77 µM [^14^C]creatine for 20 min at 37 °C and washed. Efflux transport was initiated by the addition of a transport solution. The cells were incubated at 37 °C or 4 °C for designated time periods. The transport was terminated by sampling the transport solution and washing the cells. The efflux ratio was calculated using Eq. (2). The knockdown analysis of MCT12 in TR-CSFB3 cells were performed as described previously [14].

2 $${\text{Efflux}}\,{\text{ratio}}\,\left( \% \right) = \frac{{[{}^{14}{\text{C]Creatine}}\,{\text{in}}\,{\text{the}}\,{\text{medium}}\,\left( {{\text{dpm}}} \right)}}{{[{}^{14}{\text{C]Creatine}}\,{\text{in}}\,{\text{the}}\,{\text{medium}}\,{\text{and}}\,{\text{the}}\,{\text{cells}}\,\left( {{\text{dpm}}} \right)}} \times 100$$

### Lateral ventricular micro-injection

Anesthetized rats were placed in a stereotaxic frame (SR-5R; Narishige, Tokyo, Japan). The transport solution (10 μL/rat) consisting of [^3^H] d-mannitol (reference compound for diffusion; 24.4 pmol/rat) and [^14^C]creatine (877 pmol/rat) was microinjected into the left lateral ventricle. After the designated time period, CSF (50 μL) was collected from the cisterna magna, and the cerebrum, midbrain, and cerebellum were isolated. These isolated samples were dissolved in 2 N NaOH at 55 °C for 3 h. The radioactivity of these samples was then measured using an AccuFLEX LSC-7400 instrument.

The concentration–time curves of [^3^H]D-mannitol and [^14^C]creatine were fitted to a one-compartment model [Eq. (3)] using a nonlinear least-squares regression analysis program (MULTI) [18]. *C*_CSF_ and *k*_el_ indicate the concentration in the CSF and elimination rate constant, respectively. The transfer of [^3^H]D-mannitol and [^14^C]creatine from the CSF to the brain is represented by Eq. (4). The brain-to-CSF concentration ratio [*X*_*Brain*_ (*t*)/*C*_CSF_ (*t*)] was calculated using Eq. (5), which is given by Eq. (4). The area under the CSF concentration–time curve (*ACC*) was obtained by integrating *C*_CSF_ from 0 min to the designated time period. Transfer clearance (*CL*_transfer_) was determined using MULTI.

3 $$C_{CSF} \left( t \right) = C_{CSF} \left( 0 \right) \times \exp \left( { - k_{e1} \times t} \right)$$

4 $$\frac{{dX_{Brain} }}{dt}{ = }CL_{transfer} \times C_{CSF}$$

5 $$X_{Brain} \left( t \right)/C_{CSF} \left( t \right) = CL_{transfer} \times ACC_{0 \to t} /C_{CSF} \left( t \right) + V_{0}$$

### Statistical analyses

Data are presented as the mean ± standard deviation (S.D.). Statistical analyses were performed using unpaired two-tailed Student’s t-test. Differences were considered statistically significant at *p* < 0.05. With mean and standard deviations of the significant different groups, the sample size was confirmed as an appropriate size using by G*Power 3 software [19].

## Results

### Expression and localization of MCT12 in CpxEpic

**Fig. 1:**
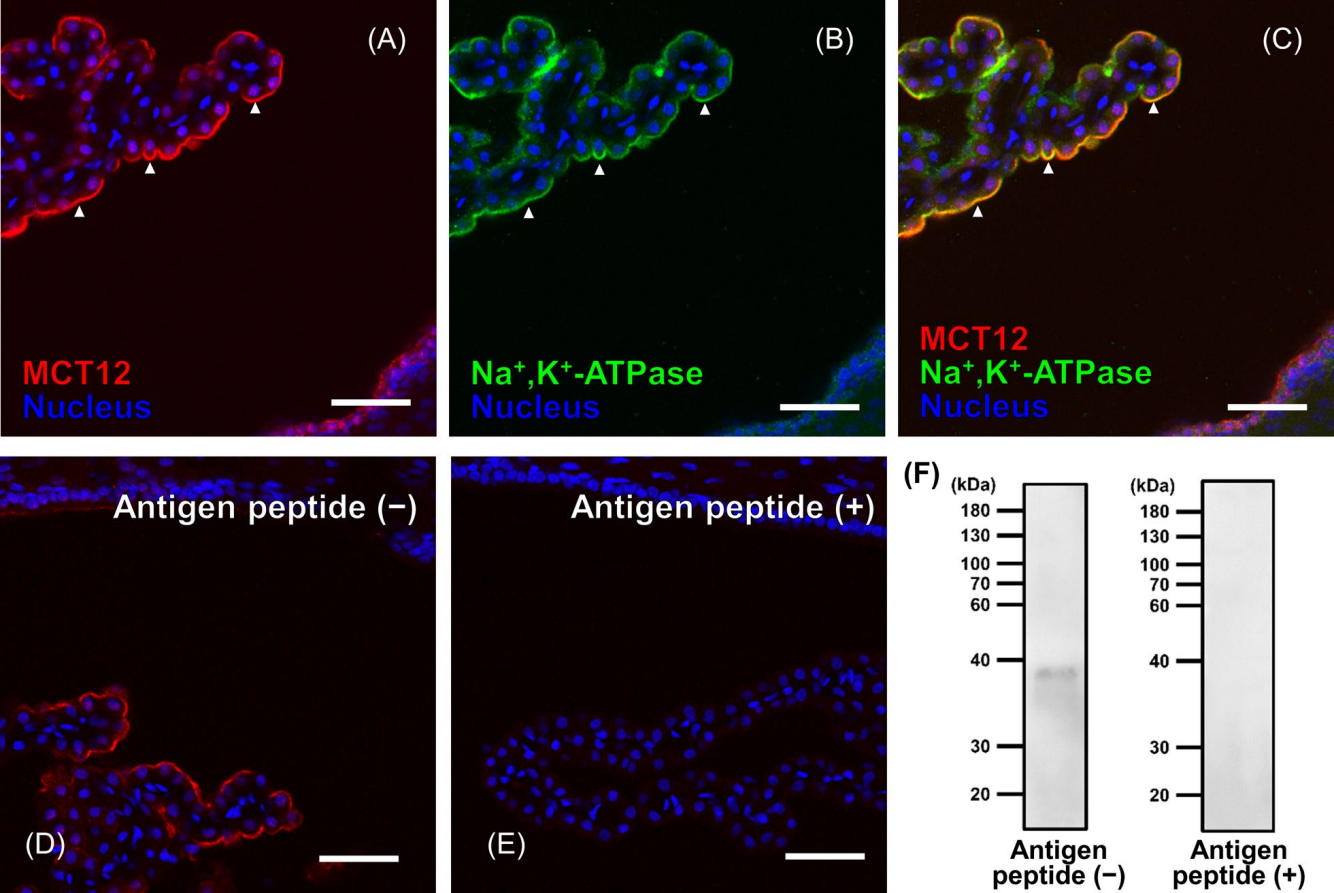
Localization of monocarboxylate transporter 12 (MCT12) in the rat choroid plexus. **A**–**C** Double immunohistochemical staining of MCT12 (red) and Na^+^, K^+^-ATPase (green) in the rat choroid plexus. Na^+^, K^+^-ATPase is used as a marker of the apical membrane of choroid plexus epithelial cells (CPxEpic). Arrowheads indicate the apical membrane of CPxEpic. **D** and **E** Immunohistochemical staining of anti-MCT12 antibodies after antigen absorption. Anti-MCT12 antibodies were incubated in PBS (−) with or without antigenic peptides of anti-MCT12 antibodies (4.36 µg/mL) for 6 h at 4 °C and then used as the primary antibodies. Nuclei were stained with 4ʹ,6-diamidino-2-phenylindole dihydrochloride (DAPI, blue). Scale bar: 50 µm. **F** Protein expression of MCT12 in CPxEpic. Anti-MCT12 antibodies were incubated in blocking buffer with or without antigenic peptides of anti-MCT12 antibodies (4.36 µg/mL) for 6 h at 4 °C and then used as the primary antibodies

Figure 1A–C show the double staining of MCT12 with Na^+^, K^+^-ATPase, which is a marker of the apical membrane of CpxEpic [20]. MCT12 immunoreactivity was observed in the choroid plexus and merged with that of Na^+^, K^+^-ATPase (Fig. 1A–C), whereas no intense immunoreactivity was observed in the brain cortex (Additional file 1). In addition, MCT12 immunoreactivity was abolished by pretreatment with antigen peptides of anti-MCT12 antibodies (Fig. 1D–E). Immunoblotting of the crude membrane of TR-CSFB3 cells using anti-MCT12 antibodies detected a signal at 37 kDa (Fig. 1F). This signal size was consistent with that detected in the study using MCT12-overexpressing cells [13]. The detected signal was abolished by pretreatment with antigen peptides of anti-MCT12 antibodies (Fig. 1F). These results confirmed the specific recognition of anti-MCT12 antibodies against MCT12 proteins. These results suggest that MCT12 is localized to the apical membrane of CpxEpic.

### Expression and activation of GAMT in CpxEpic

**Fig. 2:**
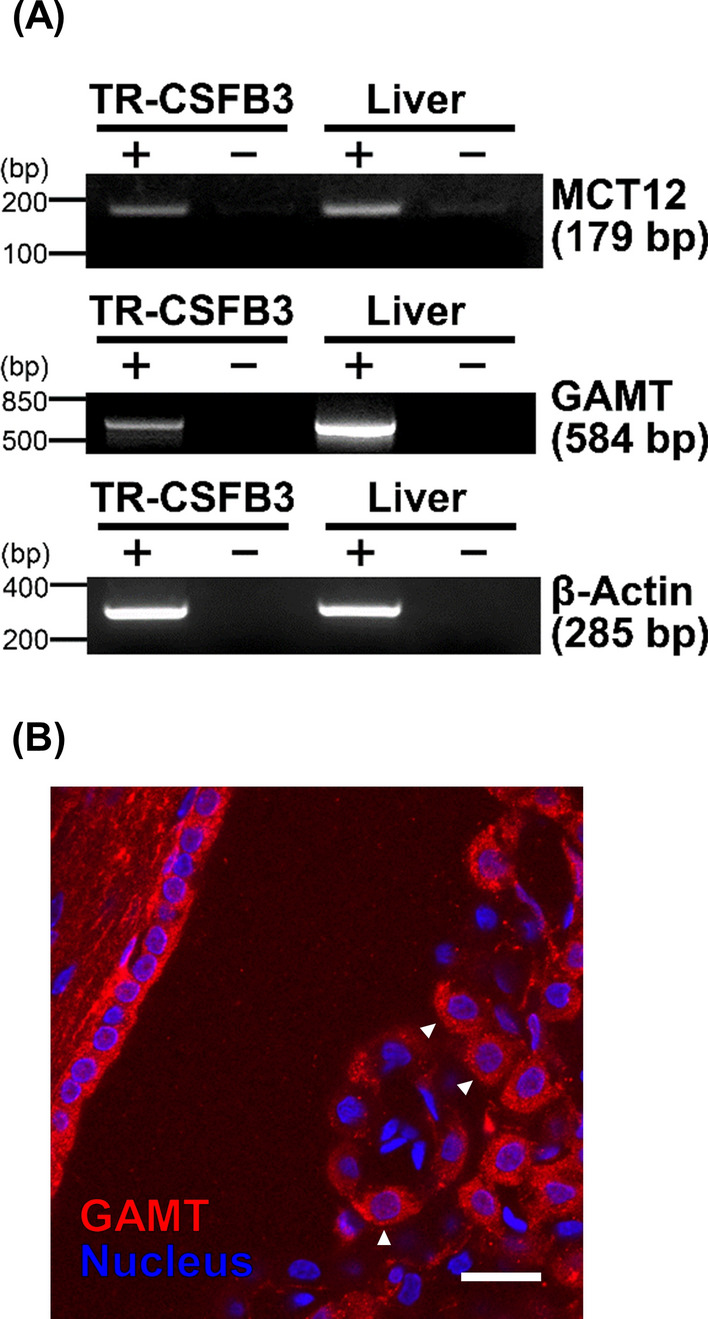
Expression of guanidinoacetate N-methyltransferase (GAMT) in CPxEpic. **A** mRNA expression of MCT12 and GAMT in TR-CSFB3 cells. Polymerase chain reaction was conducted with ( +) or without ( −) reverse transcription. The rat liver was used as a positive control. **B** Immunohistochemical staining of GAMT in the rat choroid plexus. Arrowheads indicate CPxEpic. Nuclei were stained with DAPI (blue). Scale bar: 20 µm

Considering the localization of MCT12 in CpxEpic, it is hypothesized that GAA is converted to creatine in CpxEpic, and the biosynthesized creatine is provided to the CSF via MCT12. Thus, the expression of GAMT, which catalyzes creatine synthesis from GAA, was evaluated in CpxEpic. TR-CSFB3 cells expressed GAMT mRNA in addition to MCT12 mRNA (Fig. 2A). Moreover, GAMT immunoreactivity was detected in CpxEpic (Fig. 2B, Additional file 2). Thus, GAMT expression is suggested in CpxEpic.

**Fig. 3:**
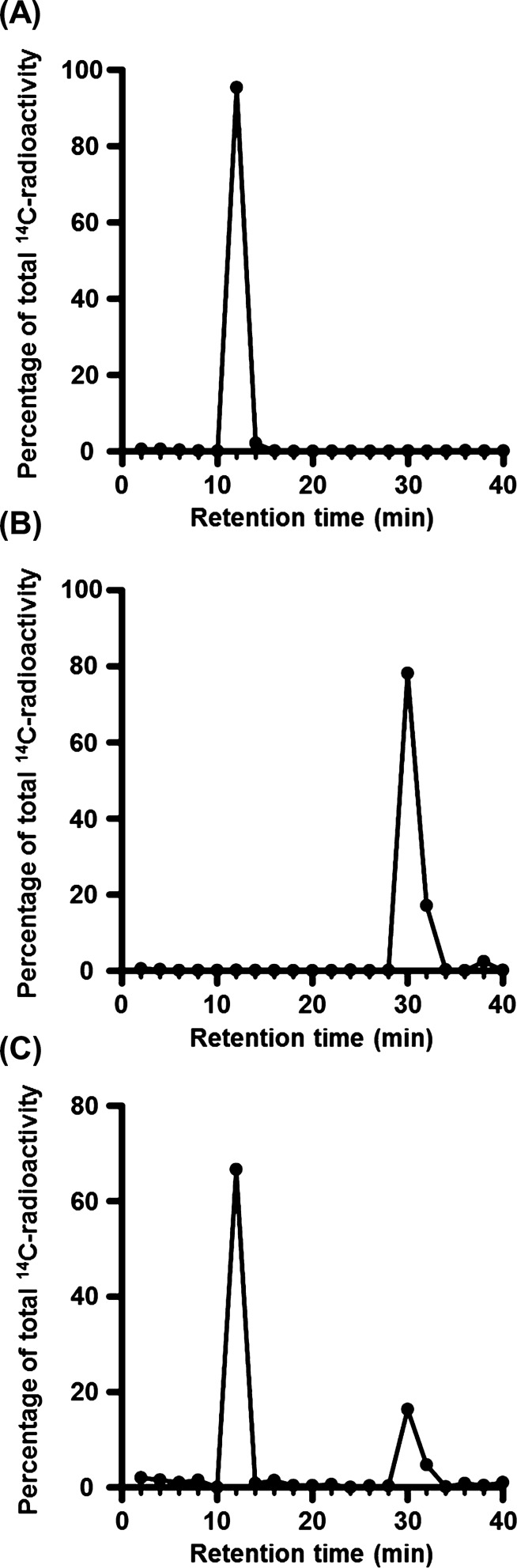
Functional expression of GAMT in TR-CSFB3 cells. Typical chromatograms of **A** [^14^C]GAA and **B** [^14^C]creatine. **C** A typical HPLC chromatogram of the cell lysate of TR-CSFB3 cells. TR-CSFB3 cells were incubated with the medium containing [^14^C]GAA (18.2 µM) for 24 h at 37 °C and the cell lysate of these cells was separated by high-performance liquid chromatography (HPLC)

Figure 3 shows functional GAMT activity in TR-CSFB3 cells. Figure 3A and B are typical chromatograms of intact [^14^C]creatine and [^14^C]GAA, respectively. Figure 3C exhibits the typical HPLC chromatogram of the cell lysate of TR-CSFB3 cells after cultivation with the medium containing [^14^C]GAA. In this chromatogram, a typical peak of [^14^C]creatine was detected in addition to that of [^14^C]GAA (Fig. 3C). Therefore, functional expression of GAMT was observed in TR-CSFB3 cells. Based on these results, GAA is suggested to be converted to creatine via GAMT after incorporation into CpxEpic.

### MCT12 contribution to the release of creatine from TR-CSFB3 cells

**Fig. 4:**
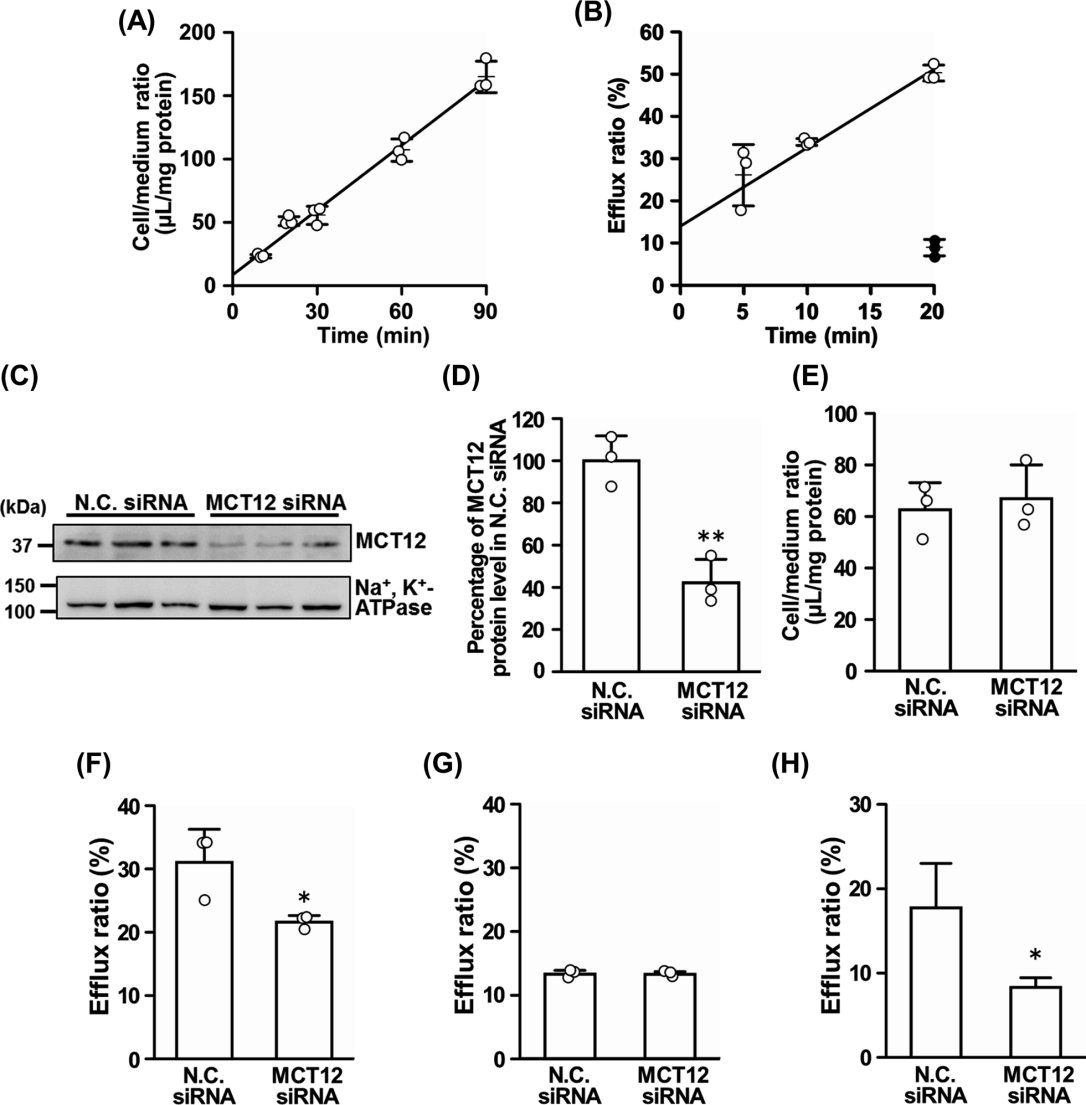
MCT12 contribution to creatine transport in TR-CSFB3 cells. **A** Time-course of [^14^C]creatine uptake by TR-CSFB3 cells. The uptake was measured for the indicated time periods at 37 °C. **B** Time-course of [^14^C]creatine efflux from TR-CSFB3 cells. After pre-incubation of [^14^C]creatine for 20 min at 37 °C, the efflux transport was measured for the indicated time periods at 37 °C (open circles) or 4 °C (closed circles). **C** Immunoblotting of the crude membrane fractions of TR-CSFB3 cells after the transfection of negative control (N.C.) or MCT12-specific small interfering RNA (siRNA). **D** Protein expression levels of MCT12 in TR-CSFB3 cells after MCT12 specific-siRNA transfection. Intensity of the signal was evaluated using the ImageJ software and the signal levels of MCT12 were normalized to that of Na^+^, K^+^-ATPase α1. **E** [^14^C]Creatine uptake by TR-CSFB3 cells after MCT12 specific-siRNA transfection. The uptake was measured for 20 min at 37 °C. **F** and **G** Efflux transport of [^14^C]creatine from TR-CSFB3 cells after the knockdown of MCT12. After pre-incubation of [^14^C]creatine for 20 min at 37 °C, efflux was measured for 20 min at 37 °C (**F**) or 4 °C (**G**). **H** Subtraction of the efflux at 4 °C (**G**) from that at 37 °C (**F**). Each column represents the mean ± standard deviation (S.D.) (*n* = 3). Each open and closed circle represents an individual data point. *p < 0.05, **p < 0.01, significantly different from the conditions of N.C. siRNA transfection

GAA is metabolized to creatine after its influx into CpxEpic. After this conversion, creatine release from CpxEpic into CSF via MCT12 is a considerable process. Thus, transport analyses were performed using TR-CSFB3 cells. Figures 4A and B show the time course of the influx and efflux transport of [^14^C]creatine in TR-CSFB3 cells, respectively. The influx transport of [^14^C]creatine into TR-CSFB3 cells exhibited a time-dependent increase (Fig. 4A), confirming the results of previous study [6]. In addition, TR-CSFB3 cells showed a time-dependent increase in the efflux transport of [^14^C]creatine until at least 20 min after pre-incorporation of [^14^C]creatine (Fig. 4B). The efflux transport decreased at 4 °C (Fig. 4B, closed circles). To determine the involvement of MCT12 in creatine transport in CpxEpic, [^14^C]creatine transport was evaluated after MCT12 knockdown using MCT12-specific siRNA. Reduction of MCT12 protein expression was confirmed 48 h after treatment with the negative control (N.C.) or MCT12 siRNA (Fig. 4C). After comparing the signal intensities of the immunoblot after N.C. and MCT12 siRNA treatment, MCT12 protein expression levels were decreased by 57.8% in MCT12 siRNA-treated cells (Fig. 4D). The influx transport of [^14^C]creatine was barely altered by MCT12 knockdown (Fig. 4E). In contrast, the efflux transport of [^14^C]creatine from MCT12 siRNA-treated TR-CSFB3 cells decreased by 30.6% compared to that of N.C. siRNA-treated TR-CSFB3 cells (Fig. 4F). In efflux transport of [^14^C]creatine, the interception of the regression line in Fig. 4B cannot be neglected for the efflux ratio of [^14^C]creatine. As it has been reported that carrier-mediated transport, including facilitated transport, is almost abolished at 4 °C, and creatine is hardly transported by passive diffusion [6, 8, 13, 21], the interception is considered to be increased by factors, such as the adsorption of [^14^C]creatine on the cell culture plate and cell surface. To remove adsorption factors, the values of [^14^C]creatine efflux ratio at 4 °C were measured and subtracted from the values at 37 °C (Figs. 4G and H). Figure 4G shows the corrected values of the efflux ratio. The efflux ratio in MCT12 siRNA-treated cells decreased by 53.2% compared to that in the N.C. siRNA-treated cells (Fig. 4H). Considering the reduction in MCT12 protein expression by the knockdown, the contribution ratio of MCT12 in creatine efflux transport from TR-CSFB3 cells was calculated as 92.0% (= 53.2/57.8 × 100). Therefore, MCT12 suggestively contributes to the release of creatine from CPxEpic.

### In vivo CSF-to-brain parenchyma transfer of creatine after intracerebroventricular injection

**Fig. 5:**
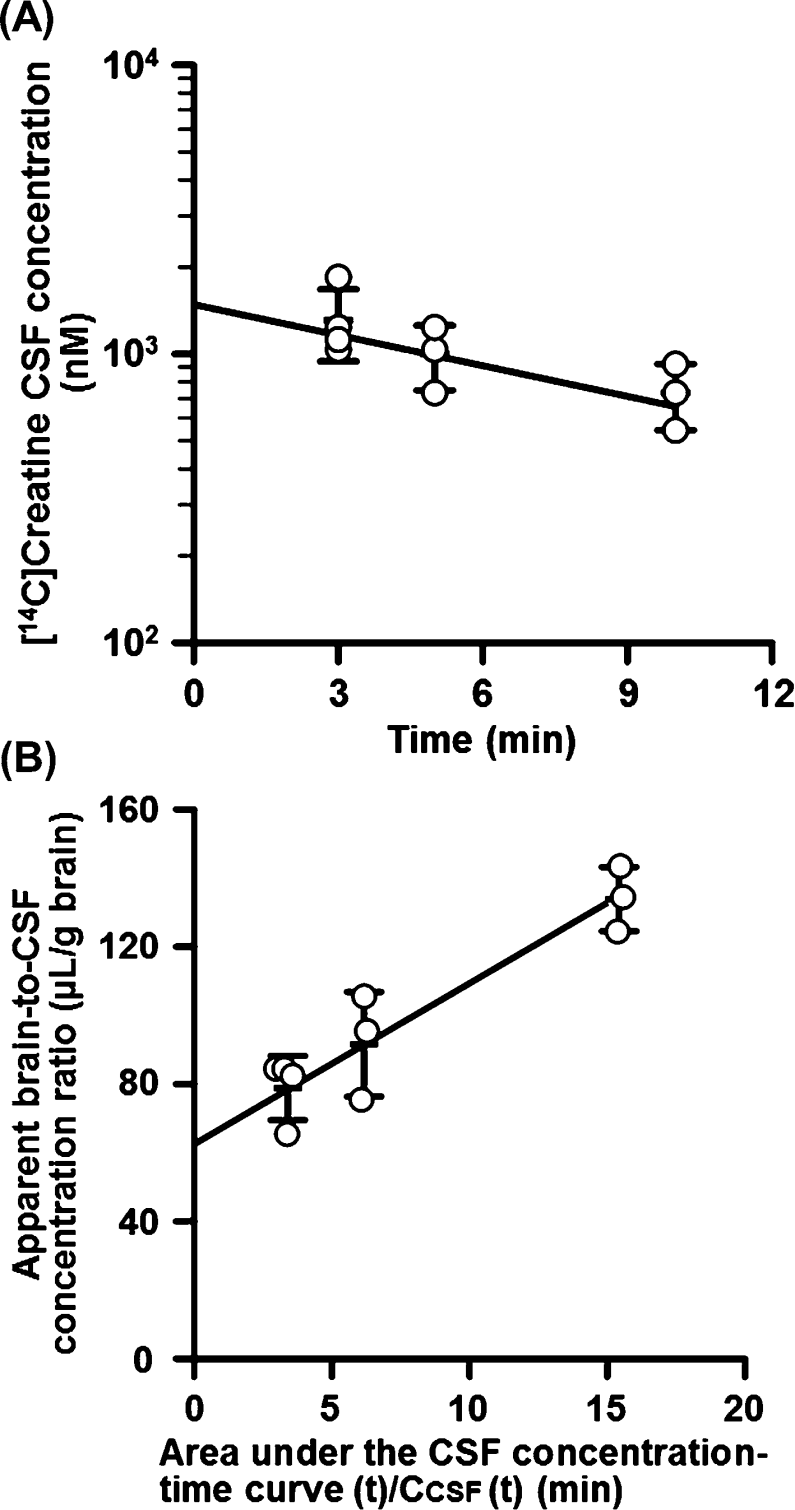
In vivo cerebrospinal fluid (CSF)-to-brain parenchyma transfer of [^14^C]creatine after intracerebroventricular injection. **A** Time-course of [^14^C]creatine concentration in the CSF after intracerebroventricular administration. The residual CSF concentration is the percentage of the dose remaining in 1 mL CSF. **B** CSF-to-brain parenchyma transfer of [^14^C]creatine after intracerebroventricular injection. An extracellular fluid buffer containing [^14^C]creatine (877 pmol/10 µL) was directly injected into the CSF. The solid line was fitted using a nonlinear least-squares regression analysis program. Each open circle represents an individual data point. Each central bar represents the mean ± S.D. (*n* = 3)

Based on the results of MCT12 localization and contribution to creatine release, MCT12 is suggested to contribute to creatine release from CPxEpic into the CSF. After release from CPxEpic, it is hypothesized that biosynthesized creatine is supplied to the brain parenchyma, as it shows a high demand for creatine [4, 5]. To determine the CSF-to-brain transfer of creatine, rat brain and CSF were collected after the intracerebroventricular administration of creatine. After intracerebroventricular injection of [^14^C]creatine, its concentration in the CSF was reduced at each time point for up to 10 min (Fig. 5A). In contrast, the apparent brain/CSF concentration ratio of [^14^C]creatine increased after intracerebroventricular administration, indicating the transfer of [^14^C]creatine from the CSF to the brain parenchyma (Fig. 5B). The apparent brain influx clearance of [^14^C]creatine from the CSF per gram brain (*CL*_app, CSF-to-brain transfer_) was determined to be 4.65 ± 0.89 µL/(min·g brain). In addition, the *CL*_app, CSF-to-brain transfer_ of [^3^H]D-mannitol, which is used to estimate transfer through the intercellular space, was determined to be 5.18 ± 1.91 µL/(min·g brain). Moreover, at the longest time point (Fig. 5B), more than 50% of the total administered [^14^C]creatine was detected in the brain sample.

## Discussion

Based on the results of our study, the GAA dynamics in CPxEpic were as follows: First, GAMT catalyzes the conversion of GAA to creatine in CPxEpic (Figs. 2 and 3). Second, creatine is released from CPxEpic into the CSF via MCT12 (Figs. 1 and 4). Finally, released creatine is supplied to the brain parenchyma (Fig. 5).

As GAA is known as an endogenous convulsant, the concentrations of GAA are maintained at a lower level in the human CSF (0.036–0.22 µM) than that in the plasma (0.35–3.5 µM) [6, 22]. We have revealed that GAA is removed from the CSF by incorporation into CPxEpic [6]. In this study, along with the elimination of GAA, the choroid plexus was suggested to participate in the provision of creatine to the brain. Moreover, the protein expression and activity of GAMT were detected in CPxEpic (Figs. 2 and 3). In addition, the majority of creatine was released via MCT12, which is predominantly localized on the CSF side membrane of CPxEpic (Figs. 1 and 4). Creatine in the CSF was transferred to the brain parenchyma (Fig. 5). In the brain, astrocytes, oligodendrocytes, and olfactory ensheathing glia express GAMT and are considered to be creatine-supplying cells [17]. From the results of this study, CPxEpic are considered to be creatine supplying cell in the brain, as well as these glial cells. Furthermore, the creatine concentration in the brain parenchyma is lower in patients with choroid plexus tumor (1.2–1.3 mM) compared to the normal range (3.9–6.0 mM) [23–25]. Meanwhile, the GAA concentration in the brain parenchyma is higher in patients with choroid plexus tumor (3.0–3.1 mM) compared to the normal range (0.06–0.85 mM) [23, 25]. These reports support the importance of the choroid plexus in maintaining the GAA and creatine homeostasis in the brain.

Our study suggests that MCT12 functions in releasing creatine from CPxEpic, although it can recognize GAA as a substrate and has the ability of influx transport of creatine [13]. By biosynthesis of creatine in CPxEpic, its concentration is considered to be higher in CPxEpic than that in the CSF. Since MCT12 reportedly transports substrates along a concentration gradient [13], it is considered to function as an efflux transporter of creatine in CPxEpic. Comparing MCT12-mediated transport of creatine with that of GAA, the affinity of MCT12 for creatine is approximately tenfold greater than that for GAA [13]. Thus, MCT12 is suggested to predominantly recognize creatine and function as an efflux transporter of creatine in CPxEpic. Regarding the influx transport of GAA into CPxEpic, we found that GAA uptake by TR-CSFB3 cells and isolated choroid plexus was decreased by three-quarters in the absence of extracellular Na^+^ [6]. As MCT12-mediated GAA transport hardly changed in the absence of extracellular Na^+^ [13], the contribution of MCT12 to GAA influx transport to CPxEpic is considered to be negligible. Taken together, MCT12 functions as an efflux transporter of the biosynthesized creatine in CPxEpic.

In vivo analysis revealed the transfer of [^14^C]creatine from the CSF to the brain (Fig. 5B). The *CL*_app, CSF-to-brain transfer_ of creatine was determined to be 4.65 µL/(min·g brain). This value was consistent with that of d-mannitol [5.18 µL/(min·g brain)]. Since d-mannitol is a reference compound for diffusion into the brain interstitial space via the ependymal layer [26], creatine is considered to mainly pass through the interstitial space of the ependymal layer in the CSF-to-brain transfer process. In addition, in the in vivo autoradiography, [^14^C]inulin injected into the lateral ventricle was reported to distribute to the entire brain [27]. Since inulin is known as a reference compound for diffusion as well as d-mannitol, creatine is possible to distribute to the entire brain from the CSF. Furthermore, the apparent brain influx clearance of creatine from circulating blood is 1.61 µL/(min·g brain). Comparing to the value of *CL*_app, CSF-to-brain transfer_ of creatine with this value, the CSF-to-brain transfer of creatine is considered to be a potential route for creatine supplementation to the brain. In the brain parenchyma, creatine is concentrated in high-energy-demanding cells, such as the neurons [3]. Therefore, creatine concentration in the brain parenchyma (3.9–6.0 mM) is much higher than that in the plasma (10–200 µM) and CSF (17–90 µM) [22, 23, 28]. Taken together, CSF-to-brain transfer of creatine is suggested to be one of the routes for creatine supplementation to the brain parenchyma.

In this study, the MCT12-mediated pathway was proposed as a route for creatine transfer from CPxEpic to the brain. Specifically, GAA is taken up by CPxEpic and converted to creatine. Subsequently, biosynthesized creatine is released into the CSF via MCT12. We have revealed that GAA was incorporated from the CSF into CPxEpic [6]. Considering the in vivo expression of GAMT proteins and apical localization of MCT12 shown in this study, creatine supplementation from GAA in the CSF to the brain parenchyma is considered to be carried out in CPxEpic in the physiological condition. Taken together, the MCT12-mediated pathway is one of the mechanisms of creatine supplementation in the central nervous system.

## Conclusions

In this study, the choroid plexus was suggested to play a role in GAA elimination and creatine supplementation. In CPxEpic, GAA is metabolized to creatine, and MCT12 mediates the release of creatine into the CSF. Through these processes, the choroid plexus may be associated with creatine homeostasis in the brain. In addition, we have suggested that CPxEpic plays a role in the elimination of d-serine, a co-agonist of N-methyl-D -aspartate (NMDA)-type glutamate receptors, from the brain by the incorporation and metabolism of d-serine [29]. Therefore, along with BCSFB constitution, the choroid plexus is proposed to have an important function in nutrient metabolism, thereby maintaining nutrient levels in the brain.

Since alternation of this MCT12-mediated creatine provision route is considered to disturb the balance of creatine and GAA levels in the brain and possibly cause epilepsy, further studies, such as in vivo MCT12 knockout, can lead to further understanding of the pathology of epilepsy and regulating seizures.

## Supplementary Information

**Additional file 1.** Immunohistochemical staining of MCT12 in the rat brain cortex. Double immunohistochemical staining was performed using anti-MCT12 antibodies (red, A and B) and anti-glial fibrillary acidic protein (GFAP) antibodies (green, B) in the rat brain cortex. GFAP is used as a marker of astrocytes. Nuclei were stained with DAPI (blue). Scale bar: 100 µm.

**Additional file 2.** Immunohistochemical staining of GAMT in the rat choroid plexus. Immunohistochemical staining of GAMT was performed in the rat choroid plexus. Nuclei were stained with DAPI (blue). Scale bar: 50 µm.

## Abbreviations

BBB: Blood–brain barrier
BCSFB: Blood-cerebrospinal fluid barrier
CCDSs: Cerebral creatine deficiency syndromes
CPxEpic: Choroid plexus epithelial cells
CRN: Creatinine
CRT: Creatine transporter
CSF: Cerebrospinal fluid
DMEM: Dulbecco’s modified Eagle’s medium
GAA: Guanidinoacetate
GABA: γ-aminobutyric acid
GAMT: Guanidinoacetate N-methyltransferase
GAT: γ-aminobutyric acid transporter
GBA: γ-guanidinobutyrate
GES: Guanidinoethanesulfonate
GGA: α-guanidinoglutaric acid
GPA: β-guanidinopropionate
GSA: Guanidinosuccinate
HEPES: N-2-hydroxyethylpiperazine-N-2-ethanesulfonic acid
HPLC: High-performance liquid chromatography
MCT: Monocarboxylate transporter
NMDA: N-methyl-D-aspartate
OCT: Organic cation transporter
PBS(-): Phosphate-buffered saline without Ca^2^^+^ and Mg^2^^+^
PVDF: Polyvinylidene
SLC: Solute carrier
siRNA: Small interfering RNA
TauT: Taurine transporter
