# Supplementary material for: Processing mechanism of guanidinoacetate in choroid plexus epithelial cells: conversion of guanidinoacetate to creatine via guanidinoacetate N-methyltransferase and monocarboxylate transporter 12-mediated creatine release into the CSF
Source: Fluids Barriers CNS. 2022 Jun 3;19:42. doi: 10.1186/s12987-022-00328-w (PMC9164341; doi:10.1186/s12987-022-00328-w)
Supplement: Supplementary file 1 — Additional file 1. Immunohistochemical staining of MCT12 in the rat brain cortex. Double immunohistochemical staining was performed using anti-MCT12 antibodies (red, A and B) and anti-glial fibrillary acidic protein (GFAP) antibodies (green, B) in the rat brain cortex. GFAP is used as a marker of astrocytes. Nuclei were stained with DAPI (blue). Scale bar: 100 µm. [file 12987_2022_328_MOESM1_ESM.pdf]

Additional file 1

Processing Mechanism of Guanidinoacetate in Choroid Plexus Epithelial Cells: Conversion of Guanidinoacetate to Creatine via Guanidinoacetate N-Methyltransferase and Monocarboxylate Transporter 12-Mediated Creatine Release into the CSF

Ryuta Jomura[1], Shin-ichi Akanuma[1], Yoshiyuki Kubo[1], Masanori Tachikawa[2], Ken-ichi Hosoya[1]

[1]Department of Pharmaceutics, Graduate School of Medicine and Pharmaceutical Sciences, University of Toyama; 2630 Sugitani, Toyama 930-0194, Japan

[2]Graduate School of Biomedical Sciences, Tokushima University; 1–78–1 Shomachi, Tokushima 770–8505, Japan

Corresponding author: Ken-ichi Hosoya
E-mail: hosoyak@pha.u-toyama.ac.jp
Tel.: +81-76-434-7505; Fax: +81-76-434-5172

Additional file 1: Immunohistochemical staining of MCT12 in the rat brain cortex

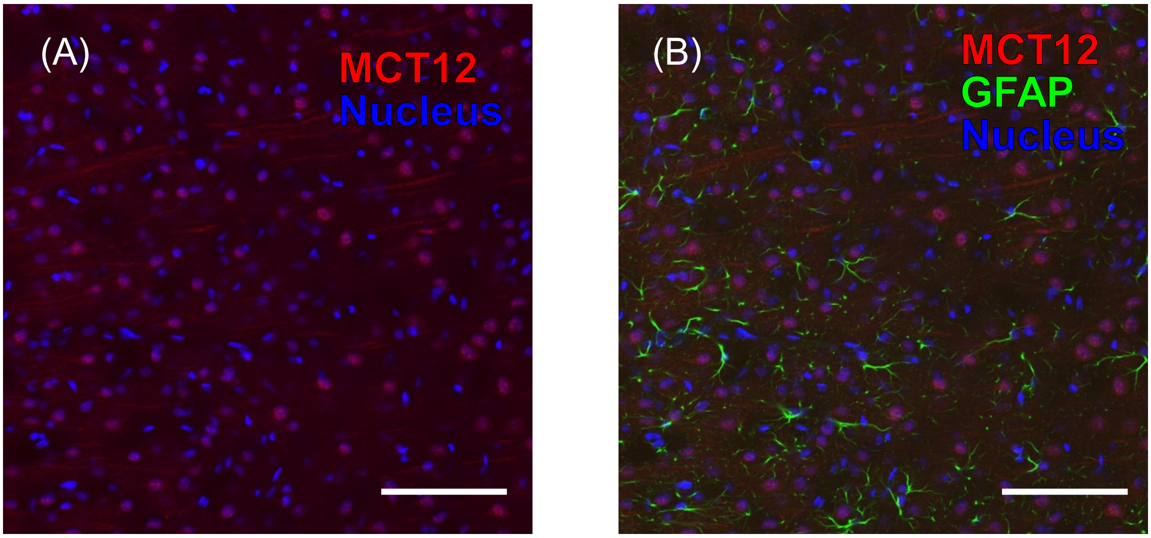

Double immunohistochemical staining was performed using anti-MCT12 antibodies (red, A and B) and anti-glial fibrillary acidic protein (GFAP) antibodies (green, B) in the rat brain cortex. GFAP is used as a marker of astrocytes. Nuclei were stained with DAPI (blue). Scale bar: 100 µm.
