# Supplementary material for: Processing mechanism of guanidinoacetate in choroid plexus epithelial cells: conversion of guanidinoacetate to creatine via guanidinoacetate N-methyltransferase and monocarboxylate transporter 12-mediated creatine release into the CSF
Source: Fluids Barriers CNS. 2022 Jun 3;19:42. doi: 10.1186/s12987-022-00328-w (PMC9164341; doi:10.1186/s12987-022-00328-w)
Supplement: Supplementary file 2 — Additional file 2. Immunohistochemical staining of GAMT in the rat choroid plexus. Immunohistochemical staining of GAMT was performed in the rat choroid plexus. Nuclei were stained with DAPI (blue). Scale bar: 50 µm. [file 12987_2022_328_MOESM2_ESM.pdf]

Additional file 2

Processing Mechanism of Guanidinoacetate in Choroid Plexus Epithelial Cells: Conversion of Guanidinoacetate to Creatine via Guanidinoacetate N-Methyltransferase and Monocarboxylate Transporter 12-Mediated Creatine Release into the CSF

Ryuta Jomura[1], Shin-ichi Akanuma[1], Yoshiyuki Kubo[1], Masanori Tachikawa[2], Ken-ichi Hosoya[1]

[1]Department of Pharmaceutics, Graduate School of Medicine and Pharmaceutical Sciences, University of Toyama; 2630 Sugitani, Toyama 930-0194, Japan

[2]Graduate School of Biomedical Sciences, Tokushima University; 1–78–1 Shomachi, Tokushima 770–8505, Japan

Corresponding author: Ken-ichi Hosoya
E-mail: hosoyak@pha.u-toyama.ac.jp
Tel.: +81-76-434-7505; Fax: +81-76-434-5172

Additional file 2: Immunohistochemical staining of GAMT in the rat choroid plexus

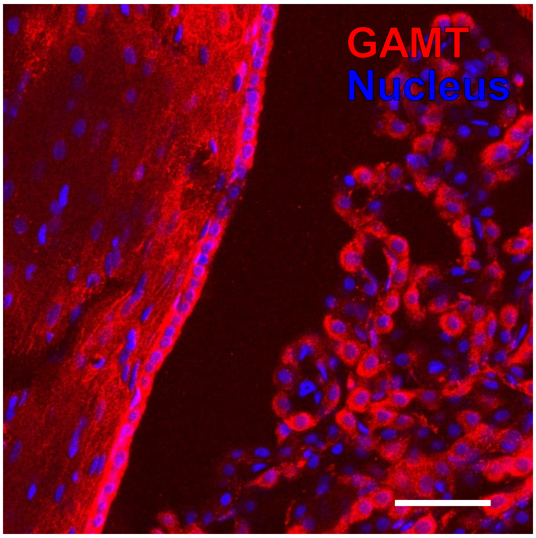

Immunohistochemical staining of GAMT was performed in the rat choroid plexus. Nuclei were stained with DAPI (blue). Scale bar: 50 µm.
